# South Korean nurses’ lived experiences supporting maternal postpartum bonding in the neonatal intensive care unit

**DOI:** 10.1080/17482631.2020.1831221

**Published:** 2020-10-06

**Authors:** Sun Young You, Ah Rim Kim

**Affiliations:** a College of Nursing, Sungshin Women’s University, Seoul, Republic of Korea; b Department of Nursing, Far East University, Chungbuk, Republic of Korea

**Keywords:** Preterm infant, neonatal intensive care units, mother-child relations, object attachment, qualitative research, family nursing

## Abstract

Purpose: Preterm birth and admission to a neonatal intensive care unit (NICU) can disrupt the parent-infant bonding relationship. Although neonatal nurses are in the best position to support maternal postpartum bonding in the NICU, few qualitative studies have described their challenges, strategies, and lived experiences. Methods: This study aimed to explore and understand the experiences and perspectives of nurses supporting infants hospitalized in the NICU and their families in relation to the bonding process. We conducted a qualitative study using interpretive phenomenological analysis with 12 in-depth, semi-structured interviews recorded and transcribed verbatim between April and November 2018. We thematically analysed the data using NVivoTM software. Results: Two themes emerged: (1) Being a bridge between separated mothers and infants (five subthemes); (2) Challenges in providing supportive care for maternal postpartum bonding in the NICU (three subthemes). Conclusions: Nurses have a variety of experiences regarding maternal postpartum bonding; however, the clinical reality of NICUs limits support for bonding formation. Although nurses face challenges (e.g., institutional policies, insufficient resources, training) when supporting maternal postpartum bonding, they act as the bridge between mothers and infants, becoming advocates for NICU families and taking care of their growth and developmental needs as caregivers.

South Korea has a rising preterm birth rate (7.6% in 2016–2017) despite having its lowest recorded fertility rate (1.05; Statistics Korea, 2019). Since 2000, the Ministry of Health and Welfare has expanded the range of the preterm birth support system, including medical and economic aid for preterm infants and high-risk pregnant women, establishment and operation of neonatal intensive care unit (NICU) regional centres, and maternal-foetal intensive care unit (Ministry of Health and Welfare, 2019). However, evidence-based guidelines for preterm infant care that encourage family participation have not been established (Lee, 2016). While advances in neonatal technology have increased survival rates for low and extremely low birth weight infants, they remain at risk of physical disabilities, neurodevelopmental sequelae (e.g., cognitive and behavioural developmental disorders, socioemotional impairments), and impaired parent-infant attachment relationships due to limited interactions and separations during NICU hospitalization (Lopez-Maestro et al., 2017).

Maternal postpartum bonding, an aspect of maternal affective status towards the infant, is a psychosocial developmental process initiated when a mother develops her role of infant care. The effects of maternal-infant bonding continue throughout a child’s life (Kim et al., 2020; Kinsey & Hupcey, 2013). Bonding between the parent and preterm infant can be facilitated through protective factors, such as nurturing touch, having close proximity, accurately interpreting infant’s cues, and being responsive to infant needs (Gribble, 2016; Lavallée et al., 2017). Preterm birth and subsequent admission to the NICU hinder the normal bonding process for parent-infant dyads (Hasanpour et al., 2018; Pennestri et al., 2015; Phuma-Ngaiyaye & Kalembo, 2016). Infants hospitalized in the NICU might have a higher prevalence of disorganized attachment at 36-months-old compared to their typically developing peers (Pennestri et al., 2015). The presence of possible neurological disabilities, along with environmental stressors (high-tech medical equipment, limited interactions, prolonged mother-infant separation) have been shown to induce maternal distress including anxiety, fear, and depression (Medina et al., 2018; Ncube et al., 2016), which is associated with reduced maternal sensitivity and inadequate infant development in infants with extremely low birth weights (Gerstein et al., 2019; Neri et al., 2017). Without adequate support for the parent-infant bonding relationship during an infants’ stay in the NICU, the long-term physical, cognitive, and psycho-emotional development of the child might not be assured. Since NICU nurses are well-positioned to work with preterm infants and their parents, strategies for supporting parent-infant bonding should be implemented during the hospital stay.

In order to support the premature infant-mother bonding process, parents and nurses should establish a partnership (Hopwood, 2010; Lavallée et al., 2017). However, in the case of one NICU in South Korea, mothers were described as voluntarily maintaining a position as the weaker party throughout their infants’ treatment, without expressing complaints (this is referred to as a *Gab-Eul* relationship in Korean society, in which one member of the dyad has less power in an unequal dynamic). Further, the mothers were reported to be helplessly dependent on the medical staff with no choice but to trust them (Choi & Lee, 2018). In previous studies, mothers who gave birth to premature infants showed a tendency to become third-party observers and tried not to interfere with the medical staff despite wanting to be actively involved in the treatment of their infants (Choi & Lee, 2018; Ncube et al., 2016). Therefore, strategic interventions focusing on building partnerships through effective communication among health care providers and vulnerable NICU families needed to be identified.

Unlike in Europe and North America, where the single-family room NICU has been increasingly adopted (Lester et al., 2016; Toivonen et al., 2017), in South Korea, early separation immediately after birth, limited visitation, and reduced participation in new-born care are common in the open-bay NICU unit system. For improved NICU outcomes such as quality of care, patient safety, and nosocomial infection, supportive work environments and favourable nurse-to-patient ratios need to be assured (Beltempo et al., 2017; Lake et al., 2016). In South Korea, even an advanced general hospital that provides the best treatment and care has a higher nurse-to-patient ratio than other countries (1:3 in Japan vs. 1:2 in the USA) with each nurse needing to care for of an average of 3.6 infants (Ministry of Health and Welfare, 2019). The current NICU setting in South Korea with high nurse-to-patient ratios and the separation of parents and infants in open-bay units has created challenges for providing family-centred care. Recent evidence shows that NICU nurses play a vital role in promoting closeness and supporting parents coping with separation from their infants (Feeley et al., 2016). The formation of bonding or emotional connection with extremely preterm infants in the NICU has primarily been examined from the viewpoint of mothers after giving birth (Medina et al., 2018; Ncube et al., 2016); little research has examined nursing support for maternal postpartum bonding in NICUs in South Korea. Further, no qualitative studies have been conducted in South Korea to describe the challenges, strategies, and lived experiences of NICU care from the viewpoint of neonatal nurses.

## Purpose

To address this gap, this study aimed at exploring, describing, and understanding nurses’ experiences of and perspectives on the neonatal bonding process while working with NICU preterm or low-birth-infants and their families.

## Design and methods

### Study design

Interpretive phenomenology (Heidegger, 2010) was employed to understand: 1) nurses’ feelings about the relationship between the preterm infant and mother in the NICU; 2) what types of experience NICU nurses had regarding caring for the parent-infant dyadic relationship; 3) what supports were available and meaningful to the nurses that helped them facilitate maternal bonding to the infants; and 4) what challenges were faced when providing supportive care to NICU families. The qualitative design was deemed suitable for this study as, by moving beyond a simple description of the experience, it allowed for the interpretation of the lived experiences in a subjective context to give insight into how a specific group of people, nurses, contextually experience a specific phenomenon, providing supportive care for mother-infant bonding.

### Setting and participants

Inclusion criteria were the ability to speak Korean, working as a Registered Nurse in a NICU in South Korea, and having more than five years of clinical experience. The sample was purposefully selected, using a snowballing sampling technique to recruit nurses. Five of the participants were invited by the corresponding author for this study (colleagues from the same university, graduate school, or hospital), and others were recommended by the previously invited participants (*n =* 7). All participants were determined to have sufficient opportunities to express their care experiences fully. Participants were interviewed in cafes near their workplace or home, offices, and a conference room in the school of nursing. The final sample included transcripts from 12 NICU nurses at six tertiary hospitals in South Korea. All were women, with a mean age of 32.33 (SD = 6.55, Range = 27–50) years and a mean clinical experience of 116.02 months (SD = 58.85, Range = 71–288) in the NICU (Table I). One-third of the participants had at least one child. Four graduated with master’s degrees in nursing fields, four were Master of Science in Nursing students, and the rest had a four-year bachelor’s degree in nursing.

**Table I.** Profile of participants in field work phase (N = 12).

No.	Sex	Age	Marital Status	Having a child or children	Education Level	NICU Clinical Experience	Department	Hospital Type and Location
P1	F	32	Married	1 child	Master’s student	8 years	NICU	Tertiary hospital, Seoul
P2	F	50	Married	2 children	MSc	24 years	NICU& nursery	Tertiary hospital, Seoul
P3	F	30	Single	No	Master’s student	7 years	NICU	Tertiary hospital, Seoul
P4	F	27	Married	No	Master’s student	7 years	NICU	Tertiary hospital, Gyeonggi Province
P5	F	28	Single	No	BSc	7 years	NICU	Tertiary hospital, Seoul
P6	F	30	Single	No	MSc	9 years	NICU	Tertiary hospital, Daejeon
P7	F	33	Single	No	Master’s student	11 years	NICU	Tertiary hospital, Seoul
P8	F	28	Single	No	BSc	5 years 6 months	NICU	Tertiary hospital, Gyeonggi Province
P9	F	28	Single	No	BSc	5 years 5 months	NICU	Tertiary hospital, Gyeonggi Province
P10	F	40	Married	1 child	MSc	12 years	NICU	Tertiary hospital, Seoul
P11	F	30	Single	No	BSc	9 years 5 months	NICU	Tertiary hospital, Seoul
P12	F	32	Married	1 child	MSc	9 years	NICU& nursery	Tertiary hospital, Seoul

*Notes*. NICU, neonatal intensive care unit.

### Data collection

Data were collected from April to November 2018 by the corresponding author, who had been working as a neonatal nurse. In-depth, semi-structured interviews were performed and recorded until data saturation was reached. The researcher contacted participants more than once during the data collection to thank them and to ensure that the participant had answered the interview questions fully. Additional interviews (by phone, mail, or face-to-face) were implemented under some circumstances. For instance, one nurse wanted to share more details of her experiences, along with a few photos, and some participants were contacted to confirm the meaning of what they said. The interview was set at a time and place convenient for each nurse, and we used flexible approach with informal language during the interviews. Nurses were asked general questions about how their day was going to create a comfortable environment prior to the first interview question. Table II shows examples from the guiding questions for the interview. These questions were only used to guide interviews and were not asked in any sequence following the nurses’ responses to prior questions. The length of the interviews ranged between 45 and 110 minutes.

**Table II.** Examples of interview guiding questions.

No.	Examples
1	What do you think about bonding between mothers and premature infants in the NICU? How do you feel about that? (*or* Tell me about your impression of the bonding relationship between mothers and preterm infants in the NICU.) What supportive nursing care aids maternal postpartum bonding ? Describe the kind of nursing care needed to support bonding between mothers and preterm infants during hospitalization in the NICU. (*or* Share examples regarding how NICU mothers and their babies were supported by nursing care to promote the bonding relationship.) What trainings or education systems for supportive nursing care on the maternal postpartum bonding development are occurring in the hospital (medical centre)?
2	
3 4 5	

*Notes*. NICU, neonatal intensive care unit.

### Ethical approval

Ethics approval was granted by human research ethics committees (Ethics Approval Number ***-17-140-2). Participants were given information about the study background, and written informed consent was obtained before the start of the interview. Participants were told that they could withdraw consent at any time and that all interviews recorded would be transcribed confidentially and analysed anonymously.

### Data analysis

In-depth semi-structured interviews were transcribed verbatim and then loaded into the data analysis software package, NVivo^TM^ software, Ver 12. Each recording was listened to more than three times to assure transcription accuracy. We listened to each interview while reading the transcripts and field notes to facilitate understanding the meaning of the participants’ words and intent. The interview data were examined for information about meaning, and notes were made to reflect the nurses’ experiences adequately. After coding each interview descriptively, we grouped similarities among the nurses’ initial codes or content into descriptive themes. Subthemes from each transcript emerged and were recorded as new codes in a format that allowed for them to be categorized into new groupings within NVivo. An iterative process of developing themes and recategorizing codes was performed until the themes clearly emerged from and sufficiently reflected the data.

### Rigour

Rigour was upheld based on Lincoln and Guba criteria, including credibility, dependability, and transferability, to secure the trustworthiness of qualitative data (Connelly, 2016; Polit & Beck, 2012). Establishing credibility is the most important aspect of qualitative studies and is assured when researchers employ standard qualitative approaches or procedures during data collection and analysis (Connelly, 2016). To enhance credibility, we recruited nurses with varying levels of clinical experience in different hospitals, which increased the likelihood of gathering evidence from multiple aspects and contexts of clinical practice. Furthermore, we provided verbatim quotations from the participants. The same author performed all the interviews and coded the transcripts using a combination of open (line-by-line and word-by-word) and in-vivo coding to explore themes, concepts, and patterns (Hsieh & Shannon, 2005; Thomas, 2006). A preliminary coding book was developed, and the first author critically reviewed and refined these codes following an audio review of each interview and blinded co-coding. In order to acknowledge and minimize biases as well as generate codes and categories reflecting data, the authors had ongoing discussions and kept an audit trail of the coding and decision processes for the interpretations of findings throughout the analysis. Consistent engagement with nurses, member-checking (agreement among co-researchers, experts, and participants regarding the study findings), and self-description/reflexivity (discussion of the researchers’ position within the study and maintenance of a reflective journal to recognize and reduce any personal biases) were techniques used to increase credibility. To establish the dependability of this study, we recorded interviews and documented our research activities, including any events (what we observed) and decisions about the study (whom to interview and when). To support transferability, we provided a clear and detailed description of participants, locations, context, data collection, and data analysis.

## Results

Data analysis identified two themes related to the nurses’ lived experiences supporting maternal postpartum bonding in the NICU: (1) being a bridge between separated mothers and infants and (2) challenges in providing supportive care for maternal postpartum bonding in the NICU (Table III).

**Table III.** Themes, subthemes and units of meanings.

Theme	Subtheme	Units of meaning
Being a Bridge between Separated Mothers and Infants	Understanding the mothers’ feelings and emotions	Negative feelings (feelings of guilt, loss, numbness, hopelessness, ambivalence, and desperation), remaining uninvolved with caregiving, care responsibility for other children, communication difficulties, a multicultural family, social culture of patriarchal Confucianism, the lack of familial support
	Becoming advocates for NICU families	Physical separation, infant-mother connection, a voice for realization of mother-baby’s bonding and families, helping mother’s decision-making, sharing information, detailed explanation, consideration of the mother’s perspective
	Facilitating mother-infant interactions and physical contact	Parental touch (e.g., massage, kangaroo care) or hugs and talk, individualized space, memorable or special things mothers made, feeling connected to the infants
	Providing care for infants as a temporarily designated mother	24-hour caregiving on behalf of the mother, feeding, diaper change, bathing, providing care, approved or accepted roles, primary caregivers after discharge, follow-up after discharge
	Finding strategies to work with mothers as a partner in infant care	Establishing a rapport, a cooperative relationship, emotional support, empathy, positive expressions and words, attentive listening, sharing unique information, explanation of treatment, prenatal consultations, self-help groups, art therapy, laughter therapy, counselling, parenting education
Challenges in Providing Supportive Care for Maternal Postpartum Bonding in the NICU	Inefficient hospital systems and policies	Institutional system or policy, human resource/nurse staffing level, specialized workforce, sharing cases of mother-infant bonding, limited visitation policy, disease treatment-focused
	Attempts to provide individualized and tailored approaches for families	Similar to the mother’s womb, maternal voice, family’s point of view, allow family items (e.g., photos, mobiles), role of caregiver, positive memories (e.g., a 100-day party, journals, a mobile platform/application for childcare), consideration of family preferences or priorities
	Lack of training, education, and guidelines for nurses	Lack of awareness of the importance of bonding, education and training for nurses, training on therapeutic communication, professional skills (e.g., conversation, breastfeeding skills), repetitive training of therapeutic and basic care, based on the parents’ needs

*Notes*. NICU, neonatal intensive care unit.

**1. Being a Bridge between Separated Mothers and Infants**

On behalf of mothers, NICU nurses stay with infants around the clock and serve as the bridge between physically separated mothers and infants. The NICU nurses we interviewed faced complex emotions and thoughts regarding the maternal role and the reality of the NICU, which provided limited support for maternal postpartum bonding. Nevertheless, the nurses had faith in their experience and attempted to promote maternal postpartum bonding; the nurses were particularly interested in evidence-based nursing interventions to improve maternal postpartum bonding in the NICU.

**1–1. Understanding the mothers’ feelings and emotions**. The nurses shared that unexpected preterm birth led to the mother being isolated from her baby and the experience of emotional distress, including feelings of guilt, loss, numbness, hopelessness, ambivalence, and desperation. The process of maternal bonding with the infant might initially begin with negative feelings, which could delay both the mother’s acceptance of the premature baby as being her own and her being immersed in her role as a mother. Because of their vulnerable emotional states and the physiological instability of the infants, mothers experienced difficulties feeling like mothers.

There is a sense of guilt inside the mothers, so they used to cry during visiting hours. Sometimes they ask questions that nurses cannot quite understand, which seems to be caused by their not accepting the current situation. This may make it hard for them to form a bond with their infant. There seems to be an ambivalence of sorts. They know they must love their child, but they are too scared to love. (P2)

**1–2. Becoming advocates for NICU families**. The NICU admission/discharge of premature infants involves transformational changes in the development of maternal postpartum bonding. Being at the infants’ bedsides 24 hours a day in the NICU, nurses are ideally placed to understand infants’ health conditions and share information with mothers and families. The nurses we interviewed also tried to provide detailed explanations about the infants’ condition in consideration of the mothers’ perspective, to reduce their anxieties and concerns.

I [the nurse] always wonder if what I told the mother [of a premature infant] helped her. I said in a positive manner, ‘It is not your fault. The baby must have been so eager to see you. The baby is relatively healthy’. (P1)

The medical staff can have conversations with the parents to encourage the mothers to develop bonding. Telling them to cheer up and be brave … (P3)

For high-risk mothers who are likely to give birth to infants with very low birth weight, I provide an explanation of how the premature infant care will proceed through prenatal consultations conducted by the maternity clinic and the neonatal clinic. (P2)

**1–3. Facilitating mother-infant interactions and physical contact**. Mothers of premature infants have insufficient opportunities for natural contact with their new-borns, and thus they have difficulty establishing maternal postpartum bonds with their infants. Under such circumstances, the nurses we interviewed promoted and encouraged interactions between mothers and infants and encouraged the mothers to visit the NICU frequently. When mothers faced challenges with physical contact with their infants because of long-distance visitation or, when mothers were at high risk, hospitalization, the nurses called them and sent photos or videos.

I encourage the mothers to keep in contact with the infants as much as possible, and I tell them to pump and bring the breastmilk to the NICU. (P3)

**1–4. Providing care for infants as a temporarily designated mother**. The NICU medical environment provides professional treatment, care, and nursing for high-risk or premature infants. New-born care such as feeding, diaper changing, and bathing, which traditionally takes up a sizable amount of the mother’s time, is handed over to NICU nurses. In our study, the mothers of premature infants approved or accepted the role of the nurses as temporarily designated mothers who provide care on the mothers’ behalf. The nurses were aware of their role as a temporarily designated mother.

Some mothers called us ‘nurse moms’. They told us that their babies were growing well and were so healthy thanks to the good care of us nurse moms. (P1)

**1–5. Finding strategies to work with parents as partners in infant care**. NICU nurses and mothers are important partners in achieving the goal of “optimal growth and development of the infants”. Nurses and mothers maintain a partnership and even companionship during premature infants’ care by supporting and interacting with one another and forming mutual trust through communication. In our study, nurses shared unique information, such as details about infants’ characteristics, with the mothers to relieve their vague anxieties and emotional detachment.

For the nurses, the skill to form a rapport with the mothers and fathers is very important. It is important to tell them as much as you know about the infants, hear them out, and sympathize with them. (P4)

**2. Challenges in Providing Supportive Care for Maternal Postpartum Bonding in the NICU**

In this NICU, nurses faced various challenges in supporting the development of mother-infant bonding. Above all, the nurses thought that establishing 24-hour autonomous visiting systems and hiring sufficient medical staff were essential prerequisites. Thus, a stable system and improved ward policies needed to become priorities at the institutional level in the hospital. In addition, understanding the mothers’ preferences as important subjects in the bonding relationship and creating tailored approaches to meet these preferences were needed. The nurses developed measures to create a family-centred therapeutic care environment rather than a treatment-centred environment. Providing education, training, and guidance on mother-infant bonding was believed to help actualize the needed nursing support for the relationship between premature infants and their mothers in the NICU.

**2–1. Inefficient hospital systems and policies**. During the infants’ NICU stay, the nurses encountered a variety of barriers related to relationship-based care, including the institutional system, staffing levels, and human resources, as well as maternal health problems. As NICUs in South Korea operate on a three- or two-shift system, changing staff every 8 or 12 hours, it is hard for the same nurses to care for the same infants and parents regularly to help support mother-infant bonding. Infants need such stable care, and nurses need to build a partnership with mothers based on communication and trust.

A 24-hour autonomous visiting policy and breastfeeding in the NICU all sound good. However, in order to make it come true, the hospital system has to be able to support it. (P5)

The number of infants that doctors and nurses are in charge of should be reduced. This will allow them the time to talk to the parents. (P2)

Due to the nature of the hospital, in the NICU, the nurses keep changing several times a day, which could be emotionally confusing for the babies. It may be difficult for them to form an attachment to fixed subjects. It would be a good idea to provide somewhat consistent nursing care. (P7)

**2–2. Attempts to provide individualized and tailored approaches for families**. The NICU is a professional medical environment designed to promote the growth and development of infants, artificially replacing the maternal uterine environment. Therefore, the NICU environment should be tailored to the infant’s point of view to be similar to the mother’s womb. For NICU families, the opportunities for adapting to the role of caregiver and developing a bonded relationship with the infant should be provided from immediately after birth until being discharged from the NICU. A variety of strategies that effectively reflect families’ needs must be considered, such as providing access to the mother’s familiar voice, displaying family photos, and providing mobiles for stimulation of the infant’s senses while reducing stress.

The mothers have brought mobiles, black-and-white baby books, family photos, and silver bracelets. … The things that the families bring may cause trouble (as a source of infection), but I think they help with forming a good relationship. (P4)

The participants developed measures to support maternal postpartum bonding in the NICU, including helping mothers take continuous interest in their infants, supporting and encouraging maternal roles, and creating positive memories for the mothers with their infants. Such measures included calligraphic art describing the unique and individual characteristics of each infant, 100-day parties for the infants and families, rolling papers, sharing photo albums, and joint journaling between the mothers and the nurses using a mobile platform (a childcare application).

When an infant who had been born with 530g bodyweight reached about 3000g bodyweight, I gave the family a piece of calligraphy drawing saying, ‘Slow and Steady’ Those few letters made the mother so happy. (P4)

**2–3. Lack of training, education, and guidelines for nurses**. The participants reported that not only the lack of standard guidelines and protocols for providing supportive care for bonding relationships in the NICU but also the lack of awareness by administrators and physicians of the importance of bonding relationships hindered changes in care. Some nurses wondered about the long-term effects of supportive care on the relationship in preterm infant-mother dyads and emphasized the importance of a consensus regarding the facilitation of this vulnerable relationship as a focus of health providers and health policymakers when providing supportive care to foster bonding relationships in the NICU.

The training in the NICU seems to focus on medical aspects only. For nurses to establish their identity as professionals and study the essence of nursing, professional training is required focusing on attachment, interactions, and family. (P7)

Regarding education and training for nurses, training on communication with the parents of premature infants was in high demand. New nurses (especially unmarried nurses) lacked direct experience with maternal postpartum bonding and motherhood. They felt they were not qualified enough to provide psychological and emotional support or to conduct education on breastfeeding.

There are certain ways of communication that mothers appreciate and feel comfortable with. It is supposed to be important to provide emotional support. I often doubt if I am communicating properly because I don’t know how to communicate in the right way. (P8)

## Discussion

This hermeneutic phenomenological research aimed to illuminate the roles and challenges faced by NICU nurses in support of maternal postpartum bonding, bridging the separation or alienation between mothers and infants. First, participants were aware of negative emotions such as guilt, loss, ambivalence, apathy/indifference, and helplessness experienced by mothers and expressed feelings of responsibility for addressing the mothers’ distress. Previous studies have shown that mothers with new-borns in the NICU were overwhelmed by shock and guilt about hospitalization, intimidating medical devices, the NICU environment, and the actual appearance in contrast to the imagined appearance of their infants (Choi & Lee, 2018). In our study, NICU nurses who were more skilled or experienced in childbirth seemed to be particularly concerned about and sympathetic to these negative feelings in NICU mothers. Some participants reported that the mothers’ initial negative emotions changed to relief, joy, or gratitude over time or the progression of treatment. These findings are consistent with previous research that reported parents’ NICU experience as being an “emotional rollercoaster” (Rossman et al., 2017; Stacey et al., 2015). Therefore, as a means of addressing the symptoms of distress that have shown to be negatively associated with maternal postpartum bonding (Bonacquisti et al., 2019; Kim et al., 2020), NICU nurses and health providers should stop viewing parents as caregivers visiting patients (Phuma-Ngaiyaye & Kalembo, 2016; Sabnis et al., 2019) and see them as targets for psychosocial intervention. The provision of information on preterm infant development and social support systems (Hopwood, 2010; Lavallée et al., 2017) such as peer breastfeeding counsellors or fellow mothers with NICU experience (Rossman et al., 2017) would be beneficial to promoting the resilience of NICU mothers with mental health needs.

One participant shared the experience of caring for a 44-year-old first-time mother who had given birth to a preterm infant. This mother lacked family support. Women of advanced maternal age (40 years or older) are at increased likelihood of experiencing adverse neonatal health outcomes such as lower birth weight, small size for gestational age, and lower Apgar scores as well as increased risk of obstetric outcomes (Sydsjö et al., 2019). Therefore, older mothers may require additional childbirth recovery time and more emotional support from families and friends compared to younger mothers. However, South Korea’s traditionally patriarchal Confucianist society might tend to blame mothers for giving birth prematurely. These beliefs can increase maternal guilt and may have a negative impact on support from the family, the fundamental social unit in South Korea. Nurses must assist individual mothers from diverse backgrounds and circumstances to cope with the stress and anxiety inherent in their infants’ NICU hospitalization through targeted therapeutic interventions during regular infant care. Nurses should encourage mothers to participate in social events such as self-help groups and family camps that allow them to exchange information and find mutual support.

Our nurse participants supported the maternal postpartum bonding process by becoming advocates for vulnerable families with preterm infants in the NICU. From an early stage of an infant’s NICU hospitalization, nurses may play the role of “attacher” between mother and infant, considering the shared needs of both (Fishering et al., 2016; Karl et al., 2006). Following the separation of parents and infants, the encouragement of close proximity such as frequent reunions, breastfeeding, physical contact, and involvement in infant care should be emphasized. In addition, while positive touch, stimulation, and skin-to-skin contact are known to enhance bonding (Feeley et al., 2016; Fishering et al., 2016), actual NICU clinical practice in South Korea allows for limited interactions due to inconsistent hospital policies and guidelines (e.g., insufficient conditions for breastfeeding and kangaroo care, limited visitation) and staffing issues (e.g., insufficient nursing staff and lack of experience or training). Despite these hurdles, responsible nurses and unit managers took the lead, through continuous communication and discussions regarding the roles of parental care, in creating a flexible and family-friendly NICU culture.

The nurses in this study were surrogate caregivers who provided continuous care for infants on the parents’ behalf and acted as teachers with therapeutic care knowledge and skills for mothers (Fishering et al., 2016; Karl et al., 2006). The NICU nurses who were experienced in childbirth seemed to be particularly sympathetic to NICU mothers’ negative feelings. However, four out of 12 of our participants had not had a child, and seven nurses had never been married; novice nurses who lacked both direct and indirect experiences of the parent-infant bonding relationship might have had limited understanding of the process of childbirth and parental bonding. Our participants expressed concern about providing parents with breastfeeding and parenting or caring skills for use at home after discharge; therefore, in-depth training and curricula in these areas need to be developed and made accessible.

The viewpoint of the participants clarified the importance of building a partnership based on the effective interaction between the NICU nurses and the mothers. A successful mother-infant bonding relationship in the NICU can be difficult to achieve without a successful relationship between parents and nurses. Thus, nurses need to appear positive, use hopeful language, and provide active mental support through attentive listening when communicating with physically and mentally vulnerable mothers (Fishering et al., 2016; Kearvell & Grant, 2010; Lavallée et al., 2017). Mutual ties and beliefs should be considered first to build a relationship based on trust with the mothers (Choi & Lee, 2018). In this study, NICU nurses identified the need for continued follow-up, self-help group formation, psychotherapy, art therapy, and education on feeding/nursing/growth and preterm infant development. These early interventions can help mothers develop the capability to cope with the traumatic shock and psychological distress of premature birth and NICU hospitalization and develop a rapport with the medical staff. A strategy in which the NICU nurses continue to communicate with the parents as equal partners in infant care and discuss their roles in achieving common goals can increase the sense of parenting efficacy and control over infant care (Brødsgaard et al., 2019). Our participants also mentioned that not only basic medical information about premature infants (such as weight gain, urine count, and test results) but also in-depth information (such as habitual positions when sleeping and unique characteristics like crying) helps parents feel close to their infants despite their physical separation. In the NICU, parents should be viewed as partners and members of the care team, rather than third parties or strangers. Parental participation in decision-making regarding infant care should be encouraged to give them a sense of control and parenting efficacy.

While nurses are in the best position to reduce the barriers that hinder their partnership with the family, a key component of family-centred care (Brødsgaard et al., 2019), institutional limitations, such as inadequate time and lack of privacy, hinder opportunities to develop this partnership. The participants in this study identified the need for improved NICU systems and policies in healthcare institutions; they saw the lack of specialized staff and systematic training courses as challenges for quality care in supporting the maternal postpartum bonding process. Study participants recognized the need to assist worried parents with ways to have physical contact and interact with their small and fragile premature infants. However, nurses mentioned that insufficient staffing as well as the absence of a private environment for training in breastfeeding and kangaroo care made it difficult to care for families. Quality care to support bonding may not be guaranteed without infrastructure improvements to staffing levels, operations, and policies, including a 24-hour accessible environment for parents and a family-centred nursing philosophy. Such systems need to be gradually implemented in South Korea, as they allow both parents to participate in infant care and promote bonding. Longitudinal investigations on the impact of familial or parental preferences and priorities on the development of parent-infant bonding are warranted in the future.

Meanwhile, education and training courses for NICU nurses regarding the promotion of maternal postpartum bonding and developmental support for premature infants should be widespread. The effectiveness of a developmental supportive care programme, which helps nurses acquire comprehensive knowledge and clinical practice for the neuroprotective development of premature infants, has been demonstrated in a recent study conducted in India (Sathish et al., 2019). Such an approach should ultimately contribute to promoting optimal growth and neurodevelopment of new-borns (Sathish et al., 2019). Therefore, active engagement with research focused on the development of such educational programmes and the evaluations of their effectiveness in domestic NICUs is needed.

According to a cross-sectional survey in Ireland, NICU staff was aware of their essential role in support of the infant-parent relationship; however, they reported insufficient education regarding the social-emotional development and bonding of premature infants as well as psychological support for the parents (Twohig et al., 2016). Therefore, nursing college curricula should include therapeutic communication courses and mental health nursing courses guided by detailed educational protocols to mitigate the psychological and emotional pain of mothers of premature infants and promote infant-mother bonding. Customized, step-by-step training courses based on NICU nursing experiences and general characteristics of nurses such as gender, age, marital status or patner’s support, and experience with childbirth, would also be beneficial.

### Implications for practice

Our findings may be useful for NICU health providers, clinicians, health system managers, and policymakers focused on an excellent understanding of how care should be provided and how the care environment in the NICU should be designed to facilitate the bonding process. In other countries, the nurse’s role in promoting maternal bonding in the NICU has been emphasized (Medina et al., 2018; Twohig et al., 2016). This role affects the social-emotional development and the holistic health of premature infants and promotes ongoing interactions between premature infants and their mothers while the infants are hospitalized in the NICU (Lopez-Maestro et al., 2017; Medina et al., 2018; Phuma-Ngaiyaye & Kalembo, 2016). Systematic education and training, proper use of professionals such as breastfeeding consultants and family counselling nurses, and strategic approaches for establishing a family-friendly physical environment in the NICU are needed. Further, hospital policies and culture should help make families feel welcome and allow nurses to demonstrate their abilities fully in support of the development of infant-parent bonding in the NICU without burnout or burdens. Active discussions about the issues related to the NICU parent-infant bonding process need to be encouraged in the medical, academic, and political sectors. Designing and improving NICU health care culture and environment to reflect the needs of both clinical practitioners and NICU families is essential.

### Study limitations

This study’s findings may have limited generalizability since this study was conducted in South Korea (i.e., several NICUs in Korean tertiary hospitals) and with a limited number of nurses. The sample mainly comprised highly educated female nurses, which may affect the transferability of the findings. This study was limited to NICU nurses’ perspectives on and experiences of the relationship between the preterm infant and the mother and did not include parents’ perspectives. Despite these limitations, we were meticulous to ensure credibility, reliability, and validity in our research approach, and this study contributes vivid experiences of nurses in supporting the premature infant-mother bonding process, identifies the gap between the theoretical knowledge of family-centred care and the domestic NICU environment, and proposes alternative measures to fill the gap.

### Conclusions

This study presents NICU nurses’ perceptions of the mother-infant bonding relationship, the role of nurses in supporting maternal postpartum bonding, the importance of building partnerships between mothers and nurses, and the challenges faced when encouraging maternal postpartum bonding. Even in the context of the South Korean NICU environment, with its limitations on conducting family-based nursing to support the bonding process, nurses saw the positive effects of maternal postpartum bonding and were interested in evidence-based nursing interventions. Nurses also came up with alternative measures, such as developing the educational curricula or protocols for premature infant breastfeeding, therapeutic communication, and nursing in support of maternal bonding. In the NICU, there is a need to nurture a family-friendly ward culture that allows nurses to play an active role in the relationship between premature babies and their mothers. Ultimately, for such changes in the environment and policies to be initiated, a paradigm shift is required towards a holistic approach that considers both premature infants and their families when providing nursing care, recognizing family members as partners critical for the healthy growth and development of the premature infant.

## Data Availability

Not Applicable
